# Case of Diseased Spleen, Successfully Treated

**Published:** 1825-10

**Authors:** Robert Hartle

**Affiliations:** Deputy Inspector of Hospitals.


### ART. III.—*Case of Diseased Spleen, successfully treated.* By ROBERT HARTLE, Esq. Deputy Inspector of Hospitals.

JOHN COLE, æt. thirteen, a native of English Harbour, Antigua, was attacked with fever in the month of May, 1824, which assumed the remittent type. The general means—free purging at the commencement, with small doses of calomel and James's powder, assisted by the liquor ammoniæ acetatis during the exacerbations, and the infusion of cinchona when the remissions were sufficiently distinct,—succeeded in removing the disease. He was, however, much reduced and emaciated; and, as the bark appeared to irritate the stomach, I gave the tinct. ferr. muriat. in doses of ten drops three times a-day, in an infusion of quassia, with nourishing diet and wine. Under this treatment he quite recovered.

In the month of February, 1825, he was again attacked by fever, which was at first severe, but terminated in intermittent. I then discovered that the spleen was enormously swelled, the margin of which extended much below the umbilicus. It had

2

a very peculiar feel, resembling that of a bladder nearly full of water: I was, in consequence, careful in examining it, and avoided making any pressure on it. The irritation produced by this enlarged viscus not only excited the tendency to fever, but no doubt deranged the chylopoietic viscera; for, unless purgatives or glysters were given, the bowels were torpid. A camphorated liniment was used to the belly every night, and the liquor arsenicalis was given in small doses, in an infusion of quassia. Under this treatment he appeared to improve, yet the spleen did not diminish in bulk; and, although he had no cold fits, the pyrexial paroxysms were more or less severe every other night.

On the morning of the 27th March, I found him free of pyrexial heat; but he complained of much pain in the spleen, particularly when I touched it. I ordered warm flannels to be applied over the belly, and an enema given immediately. I had scarcely left the house, when I was recalled, and on my return I found him in a strong convulsive fit, the muscles of the right side of his face strangely distorted, and his countenance rendered more hideous by strabismus; his pulse was not to be felt; and in an instant his extremities were cold, and covered with clammy sweat. I immediately directed my attention to the examination of the spleen, and, on removing the sheet that covered him, I was astonished to find his belly (which not ten minutes before was soft and flaccid,) tense and exceedingly distended: my conclusion, therefore, was that the spleen had burst into the cavity of the abdomen. As it was not possible to administer by the mouth, I ordered a solution of assafœtida P. L. to be given as an enema every three hours, and constant friction of warm oil over the belly and extremities. He continued in this convulsive fit, without interval, from eight o'clock in the morning until nearly nine o'clock that night, when the muscular distortion suddenly subsided, and he exclaimed "O my belly!" The pulse, which before could scarcely be felt, now gradually rose and expanded, feeling as if a tourniquet had been applied on the brachial artery, and gradually let down until the pulse had its full force. I gave him a little brandy and water, which he swallowed, and I directed a tea-spoonful to be frequently given. The assafœtida glysters were continued, a large blister applied over the belly, and the following mixture ordered:—

℞. Misturæ assafœtidæ P.L. ℥viij.

Tinct. jalap, ℥j. M. f. Cochl. magnum, unum tertia vel quarta hora.

He passed a restless night, and complained of severe pain in the belly, until four o'clock the following morning, when he had a copious watery stool, of deep yellow colour, which gave immediate relief. Fluctuation was now distinctly felt, but no trace of the spleen. I ordered the fœtid mixture, and brandy and water given in small quantities.

On the 1st April, finding very little diminution in the abdomen, I ordered ten grains of the ung. hydrarg. fort. to be rubbed on the belly every night; which was continued until the 7th, at which time the enlargement had so completely subsided, and the natural functions so much improved, that I discontinued it, fearing to saturate the system, which was already too much emaciated. Notwithstanding the diminution of the belly, and visible improvement in every respect, there remained a strong predisposition to intermittent; for every other night he was restless, with much pyrexial heat, but no cold fit.

As his appetite was very delicate, and stomach disposed to be irritable, I did not like to venture on the use of the bark; but it fortunately happened that, on the 14th of April, I received a supply of the sulphate of quinine, and, anxious to ascertain the virtue or efficacy of this new medicine, I thought him a very fit subject to try it with, and accordingly began, on the 17th April, to give two grains, dissolved in a little water, every morning fasting, and every evening at five o'clock. The night of the 18th was his fever-night; but he escaped it, and has had no return since.

I am decidedly of opinion that this case was an encysted dropsy of the spleen, and that the waters forming the tumor had burst into the cavity of the abdomen. At first I thought of tapping to evacuate the fluid; but, as he was a precarious and doubtful patient, I reflected that, if he died while under my trocar, his ignorant parent and friends would imagine that the operation had hurried his dissolution.

The assafœtida, in glysters and mixture, I gave as the best stimulant and most powerful antispasmodic. The sulphate of quinine in this case proved a powerful and permanent tonic, without any astringent qualities. I must here observe, that on the 19th I gave *one* dose of four grains, which, without quickening the pulse, excited great heat, flushed face, and anxiety; but these soon subsided by profuse and general perspiration. His appetite daily improved under the use of this medicine; the natural functions resumed their order; his strength rapidly returned; and he is now, I am happy to say, perfectly recovered. He took thirty grains of sulphate of quinine in seven days.

***Antigua; June 18th, 1825.***

